# 3-Isobutyl-4-phenyl­sulfan­yl-1*H*-pyrazol-5-ol

**DOI:** 10.1107/S1600536811002170

**Published:** 2011-01-22

**Authors:** Tara Shahani, Hoong-Kun Fun, R. Venkat Ragavan, V. Vijayakumar, S. Sarveswari

**Affiliations:** a X-ray Crystallography Unit, School of Physics, Universiti Sains Malaysia, 11800 USM, Penang, Malaysia; b Organic Chemistry Division, School of Advanced Sciences, VIT University, Vellore 632014, India

## Abstract

The asymmetric unit of the title compound, C_13_H_16_N_2_OS, contains two independent mol­ecules (*A* and *B*). The pyrazole ring [maximum deviations = 0.0049 (17) Å in mol­ecule *A* and 0.0112 (19) Å in mol­ecule *B*] makes a dihedral angle of 70.23 (11) and 73.18 (12)° with the phenyl ring in mol­ecules *A* and *B*, respectively. The isobutyl group in mol­ecule *B* is disordered over two sets of sites with a ratio of refined occupancies of 0.858 (5):0.142 (5). In the crystal, mol­ecules *A* and *B* are linked *via* a pair of inter­molecular N—H⋯O hydrogen bonds, generating an *R*_2_^2^(8) ring motif. These ring motifs are further linked into two-dimensional arrays parallel to the *bc* plane by inter­molecular N—H⋯O and weak C—H⋯S hydrogen bonds. The crystal is further stablized by weak π–π inter­actions [centroid–centroid distances = 3.5698 (13) and 3.5287 (12) Å].

## Related literature

For pyrazole derivatives and their microbial activity, see: Ragavan *et al.* (2009 ▶, 2010 ▶). For related structures, see: Shahani *et al.* (2009 ▶, 2010*a* ▶,*b* ▶,*c* ▶). For hydrogen-bond motifs, see: Bernstein *et al.* (1995 ▶). For standard bond-length data, see: Allen *et al.* (1987 ▶). For the stability of the temperature controller used in the data collection, see: Cosier & Glazer (1986 ▶).
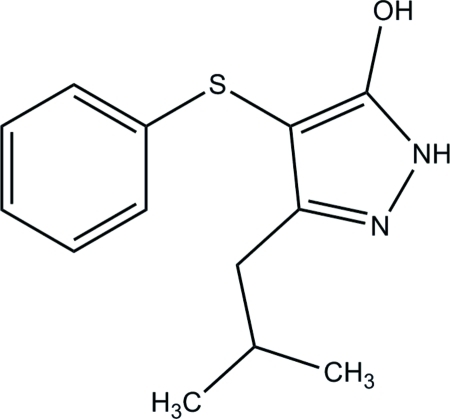


## Experimental

### 

#### Crystal data

C_13_H_16_N_2_OS
*M* *_r_* = 248.34
Orthorhombic, 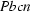

*a* = 20.7342 (6) Å
*b* = 11.1320 (3) Å
*c* = 23.1608 (6) Å
*V* = 5345.8 (3) Å^3^
*Z* = 16
Mo *K*α radiation
μ = 0.23 mm^−1^
*T* = 100 K
0.37 × 0.24 × 0.14 mm

#### Data collection

Bruker APEXII DUO CCD area-detector diffractometer
Absorption correction: multi-scan (*SADABS*; Bruker, 2009 ▶) *T*_min_ = 0.920, *T*_max_ = 0.969
33422 measured reflections
7837 independent reflections
5405 reflections with *I* > 2σ(*I*)
*R*_int_ = 0.066

#### Refinement

*R*[*F* ^2^ > 2σ(*F* ^2^)] = 0.062
*wR*(*F* ^2^) = 0.131
*S* = 1.14
7837 reflections
329 parameters
H atoms treated by a mixture of independent and constrained refinement
Δρ_max_ = 0.57 e Å^−3^
Δρ_min_ = −0.36 e Å^−3^

### 

Data collection: *APEX2* (Bruker, 2009 ▶); cell refinement: *SAINT* (Bruker, 2009 ▶); data reduction: *SAINT*; program(s) used to solve structure: *SHELXTL* (Sheldrick, 2008 ▶); program(s) used to refine structure: *SHELXTL*; molecular graphics: *SHELXTL*; software used to prepare material for publication: *SHELXTL* and *PLATON* (Spek, 2009 ▶).

## Supplementary Material

Crystal structure: contains datablocks global, I. DOI: 10.1107/S1600536811002170/lh5194sup1.cif

Structure factors: contains datablocks I. DOI: 10.1107/S1600536811002170/lh5194Isup2.hkl

Additional supplementary materials:  crystallographic information; 3D view; checkCIF report

## Figures and Tables

**Table 1:** Hydrogen-bond geometry (Å, °)

*D*—H⋯*A*	*D*—H	H⋯*A*	*D*⋯*A*	*D*—H⋯*A*
N1*A*—H1*NA*⋯O1*A*^i^	0.94 (3)	1.71 (3)	2.639 (2)	171 (3)
N2*A*—H2*NA*⋯O1*B*	0.90 (2)	1.88 (3)	2.752 (2)	161 (2)
N1*B*—H1*NB*⋯O1*B*^ii^	0.95 (3)	1.67 (3)	2.619 (2)	176 (3)
N2*B*—H2*NB*⋯O1*A*	0.85 (3)	1.91 (3)	2.731 (2)	162 (2)
C10*B*—H10*D*⋯S1*A*^iii^	0.96	2.84	3.721 (2)	153

Symmetry codes: (i) 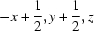; (ii) 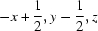; (iii) 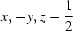.

**Fig. 1.**
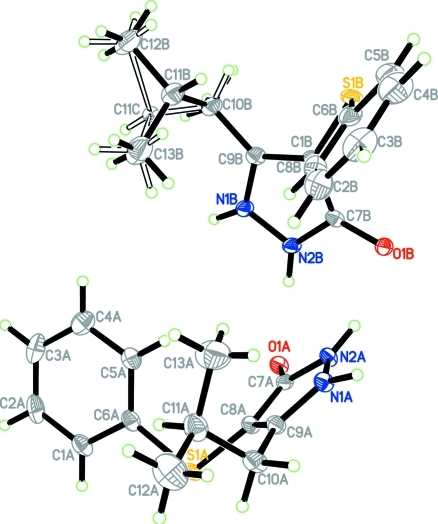
The molecular structure of the title compound, showing 50% probability displacement ellipsoids and the atom numbering scheme. The open bonds indicate the minor component of disorder.

**Fig. 2.**
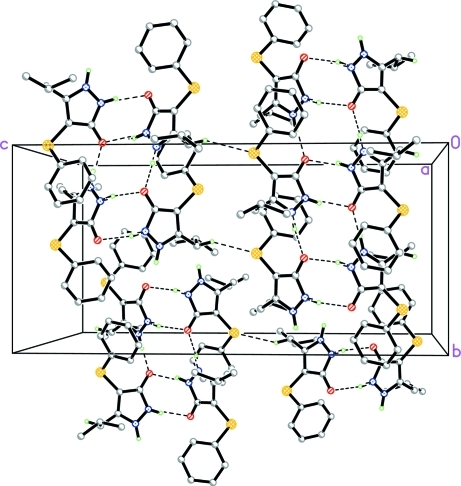
The crystal packing of the title compound, showing a two-dimensional array parallel to the bc plane. Intermolecular hydrogen bonds are shown as dashed lines.

## supplementary crystallographic information

### Comment

Antibacterial and antifungal activities of the azoles are most widely studied and some of them are in clinical practice as anti-microbial agents. However, the azole-resistant strain had led to the development of new antimicrobial compounds. In particular pyrazole derivatives are extensively studied and used as antimicrobial agents. Pyrazole is an important class of heterocyclic compounds and many pyrazole derivatives are reported to have the broad spectrum of biological properties such as anti-inflammatory, antifungal, herbicidal, anti-tumour, cytotoxic, molecular modelling, and antiviral activities. Pyrazole derivatives also act as antiangiogenic agents, A3 adenosine receptor antagonists, neuropeptide YY5 receptor antagonists, kinase inhibitor for treatment of type 2 diabetes, hyperlipidemia, obesity, and thrombopiotinmimetics. Recently urea derivatives of pyrazoles have been reported as potent inhibitors of p38 kinase. Since the high electronegativity of halogens (particularly chlorine and fluorine) in the aromatic part of the drug molecules play an important role in enhancing their biological activity, we are interested to have 4-fluoro or 4-chloro substitution in the aryls of 1,5-diaryl pyrazoles. As part of our on-going research aiming the synthesis of new antimicrobial compounds, we have reported the synthesis of novel pyrazole derivatives and their microbial activities (Ragavan *et al.*, 2009; 2010). The structure of the title compound is presented here.

In the asymmetric unit of the title compound, (Fig. 1), the 1*H*-pyrazol ring (N1/N2/C7–C9) in molecule *A* (with maximum deviation of 0.0049 (17) Å at atom N2A) makes dihedral angle of 70.23 (11)° with phenyl ring (C1–C6). The corresponding values in molecule B are maximum deviation of 0.0112 (19) Å at atom N2B and 73.18 (12)°. The torsion angles of O1/C7/C8/S1 are 5.8 (3) and 7.0 (3)° in molecules *A* and *B*, respectively. The isobutane moiety (C10–C13) in molecule *B* is disordered over two positions with refined site-occupancies of 0.858 (5): 0.142 (5). The bond lengths (Allen *et al.*,1987) and angles are within normal ranges and comparable to those closely related structures (Shahani *et al.*,2009; 2010*a*, *b*, *c*).

In the crystal packing (Fig. 2), pairs of intermolecular N2A—H2NA···O1B and N2B—H2NB···O1A hydrogen bonds link neighbouring molecules, generating *R*_2_^2^(8) ring motifs (Bernstein *et al.*, 1995). Furthermore, N1A—H1NA···O1A^i^, N1B—H1NB···O1B^ii^ and C10B—H10D···S1A^iii^ hydrogen bonds (see Table 1 for symmetry codes) link the molecules into two-dimensional arrays parallel to the *bc* plane. The crystal structure is stablilized by weak π–π interactions [*Cg*1···*Cg*2 = 3.5698 (13) Å, symmetry code, 1/2-*x*, -1/2+y, *z* and *Cg*3···*Cg*4 = 3.5287 (12) Å, symmetry code, 1/2-*x*, 1/2+y, *z*]. *Cg*1 and *Cg*3 are the centroids of the 1*H*-pyrazole rings (N1B/N2B/C7B–C9B and N1A/N2A/C7A–C9A), Cg2 and Cg4 are the centroids of benzene rings (C1B–C6B and C1A–C6A).

### Experimental

The compound was synthesized using the method available in the literature (Ragavan *et al.*,2009) and recrystallized using the ethanol-chloroform 1:1 mixture. Yield: 71%. *Mp*: 489 K.

### Refinement

The hydrogen atoms bound to C atoms were positioned geometrically [C–H = 0.93–0.98 Å] with *U*_iso_(H) =1.2 or 1.5*U*_iso_(C). The hydrogen atoms attached to the N atoms were located from the difference map and refined freely, [N–H = 0.84 (3)—0.95 (3) Å]. The isobutane moiety (C10–C13) in molecule B is disordered over two positions with refined site-occupancies of 0.858 (5): 0.142 (5). The same U^ij^ parameters were used for atom pair C11B and C11C.

### Crystal data

**Table:**

C_13_H_16_N_2_OS	*F*(000) = 2112
*M**_r_* = 248.34	*D*_x_ = 1.234 Mg m^−^^3^
Orthorhombic, *P**b**c**n*	Mo *K*α radiation, λ = 0.71073 Å
Hall symbol: -P 2n 2ab	Cell parameters from 4628 reflections
*a* = 20.7342 (6) Å	θ = 2.6–29.7°
*b* = 11.1320 (3) Å	µ = 0.23 mm^−^^1^
*c* = 23.1608 (6) Å	*T* = 100 K
*V* = 5345.8 (3) Å^3^	Block, colourless
*Z* = 16	0.37 × 0.24 × 0.14 mm

### Data collection

**Table:**

Bruker APEXII DUO CCD area-detector diffractometer	7837 independent reflections
Radiation source: fine-focus sealed tube	5405 reflections with *I* > 2σ(*I*)
graphite	*R*_int_ = 0.066
φ and ω scans	θ_max_ = 30.1°, θ_min_ = 2.0°
Absorption correction: multi-scan (*SADABS*; Bruker, 2009)	*h* = −25→29
*T*_min_ = 0.920, *T*_max_ = 0.969	*k* = −14→15
33422 measured reflections	*l* = −31→32

### Refinement

**Table:**

Refinement on *F*^2^	Primary atom site location: structure-invariant direct methods
Least-squares matrix: full	Secondary atom site location: difference Fourier map
*R*[*F*^2^ > 2σ(*F*^2^)] = 0.062	Hydrogen site location: inferred from neighbouring sites
*wR*(*F*^2^) = 0.131	H atoms treated by a mixture of independent and constrained refinement
*S* = 1.14	*w* = 1/[σ^2^(*F*_o_^2^) + (0.0338*P*)^2^ + 4.0215*P*] where *P* = (*F*_o_^2^ + 2*F*_c_^2^)/3
7837 reflections	(Δ/σ)_max_ < 0.001
329 parameters	Δρ_max_ = 0.57 e Å^−^^3^
0 restraints	Δρ_min_ = −0.36 e Å^−^^3^

### Special details

**Table:**

Experimental. The crystal was placed in the cold stream of an Oxford Cyrosystems Cobra open-flow nitrogen cryostat (Cosier & Glazer, 1986) operating at 100.0 (1) K.
Geometry. All e.s.d.'s (except the e.s.d. in the dihedral angle between two l.s. planes) are estimated using the full covariance matrix. The cell e.s.d.'s are taken into account individually in the estimation of e.s.d.'s in distances, angles and torsion angles; correlations between e.s.d.'s in cell parameters are only used when they are defined by crystal symmetry. An approximate (isotropic) treatment of cell e.s.d.'s is used for estimating e.s.d.'s involving l.s. planes.
Refinement. Refinement of *F*^2^ against ALL reflections. The weighted *R*-factor *wR* and goodness of fit *S* are based on *F*^2^, conventional *R*-factors *R* are based on *F*, with *F* set to zero for negative *F*^2^. The threshold expression of *F*^2^ > σ(*F*^2^) is used only for calculating *R*-factors(gt) *etc*. and is not relevant to the choice of reflections for refinement. *R*-factors based on *F*^2^ are statistically about twice as large as those based on *F*, and *R*- factors based on ALL data will be even larger.

### Fractional atomic coordinates and isotropic or equivalent isotropic displacement parameters (Å^2^)

**Table:**

	*x*	*y*	*z*	*U*_iso_*/*U*_eq_	Occ. (<1)
S1A	0.19888 (3)	0.02166 (5)	0.45317 (2)	0.01991 (12)	
O1A	0.28838 (7)	0.04530 (12)	0.33405 (6)	0.0167 (3)	
N1A	0.21490 (8)	0.31619 (16)	0.36323 (7)	0.0166 (4)	
N2A	0.25553 (8)	0.24348 (15)	0.33265 (8)	0.0154 (3)	
C1A	0.13701 (11)	−0.19063 (19)	0.44122 (10)	0.0229 (5)	
H1AA	0.1514	−0.2044	0.4787	0.028*	
C2A	0.10188 (12)	−0.2778 (2)	0.41238 (11)	0.0294 (5)	
H2AA	0.0922	−0.3497	0.4309	0.035*	
C3A	0.08107 (11)	−0.2593 (2)	0.35642 (12)	0.0319 (6)	
H3AA	0.0584	−0.3192	0.3371	0.038*	
C4A	0.09414 (11)	−0.1515 (2)	0.32926 (11)	0.0283 (5)	
H4AA	0.0797	−0.1386	0.2918	0.034*	
C5A	0.12885 (10)	−0.0618 (2)	0.35763 (10)	0.0216 (5)	
H5AA	0.1373	0.0108	0.3392	0.026*	
C6A	0.15065 (10)	−0.08166 (18)	0.41356 (9)	0.0189 (4)	
C7A	0.25607 (10)	0.13164 (17)	0.35621 (8)	0.0151 (4)	
C8A	0.21390 (10)	0.13713 (17)	0.40444 (9)	0.0164 (4)	
C9A	0.18970 (10)	0.25375 (18)	0.40703 (9)	0.0177 (4)	
C10A	0.14148 (11)	0.30870 (19)	0.44681 (9)	0.0222 (5)	
H10A	0.1479	0.3950	0.4477	0.027*	
H10B	0.1486	0.2783	0.4855	0.027*	
C11A	0.07172 (11)	0.2821 (2)	0.42884 (11)	0.0274 (5)	
H11A	0.0659	0.1947	0.4283	0.033*	
C12A	0.02496 (13)	0.3346 (3)	0.47288 (13)	0.0447 (7)	
H12A	0.0348	0.3034	0.5105	0.067*	
H12B	−0.0184	0.3131	0.4626	0.067*	
H12C	0.0290	0.4205	0.4733	0.067*	
C13A	0.05691 (13)	0.3300 (3)	0.36880 (12)	0.0443 (7)	
H13A	0.0122	0.3166	0.3601	0.066*	
H13B	0.0832	0.2890	0.3409	0.066*	
H13C	0.0660	0.4145	0.3675	0.066*	
S1B	0.20246 (3)	0.30672 (5)	0.09810 (2)	0.02120 (13)	
O1B	0.28972 (7)	0.28861 (12)	0.21994 (6)	0.0168 (3)	
N1B	0.21814 (8)	0.01560 (16)	0.19109 (7)	0.0166 (4)	
N2B	0.25850 (9)	0.08975 (15)	0.22121 (8)	0.0168 (4)	
C1B	0.12469 (11)	0.3859 (2)	0.18971 (11)	0.0252 (5)	
H1BA	0.1324	0.3129	0.2080	0.030*	
C2B	0.08736 (11)	0.4730 (2)	0.21669 (12)	0.0316 (6)	
H2BA	0.0703	0.4582	0.2531	0.038*	
C3B	0.07542 (12)	0.5817 (2)	0.18976 (13)	0.0364 (6)	
H3BA	0.0510	0.6401	0.2083	0.044*	
C4B	0.09985 (14)	0.6030 (2)	0.13543 (13)	0.0405 (7)	
H4BA	0.0910	0.6754	0.1171	0.049*	
C5B	0.13763 (12)	0.5173 (2)	0.10778 (11)	0.0320 (6)	
H5BA	0.1543	0.5326	0.0712	0.038*	
C6B	0.15048 (11)	0.40805 (19)	0.13536 (10)	0.0217 (5)	
C7B	0.25854 (10)	0.20082 (18)	0.19731 (8)	0.0155 (4)	
C8B	0.21715 (10)	0.19358 (18)	0.14830 (9)	0.0166 (4)	
C9B	0.19360 (10)	0.07625 (18)	0.14657 (9)	0.0176 (4)	
C10B	0.14766 (10)	0.0172 (2)	0.10600 (9)	0.0218 (4)	
H10C	0.1516	−0.0684	0.1100	0.026*	0.858 (5)
H10D	0.1596	0.0378	0.0672	0.026*	0.858 (5)
H10E	0.1699	−0.0490	0.0884	0.026*	0.142 (5)
H10F	0.1379	0.0736	0.0759	0.026*	0.142 (5)
C11B	0.07762 (13)	0.0524 (3)	0.11539 (12)	0.0241 (6)	0.858 (5)
H11B	0.0735	0.1383	0.1069	0.029*	0.858 (5)
C11C	0.0867 (8)	−0.0274 (16)	0.1281 (7)	0.0241 (6)	0.142 (5)
H11C	0.0919	−0.1125	0.1379	0.029*	0.142 (5)
C12B	0.03645 (12)	−0.0169 (3)	0.07140 (12)	0.0436 (7)	
H12D	−0.0081	0.0041	0.0762	0.065*	0.858 (5)
H12E	0.0501	0.0033	0.0330	0.065*	0.858 (5)
H12F	0.0418	−0.1016	0.0776	0.065*	0.858 (5)
H12G	0.0557	−0.0542	0.0382	0.065*	0.142 (5)
H12H	−0.0033	−0.0567	0.0805	0.065*	0.142 (5)
H12I	0.0281	0.0662	0.0632	0.065*	0.142 (5)
C13B	0.05389 (14)	0.0320 (3)	0.17556 (12)	0.0466 (8)	
H13D	0.0801	0.0763	0.2023	0.070*	0.858 (5)
H13E	0.0099	0.0581	0.1787	0.070*	0.858 (5)
H13F	0.0566	−0.0521	0.1842	0.070*	0.858 (5)
H13G	0.0806	0.0289	0.2094	0.070*	0.142 (5)
H13H	0.0454	0.1143	0.1658	0.070*	0.142 (5)
H13I	0.0139	−0.0086	0.1831	0.070*	0.142 (5)
H1NA	0.2143 (13)	0.399 (3)	0.3566 (12)	0.040 (8)*	
H2NA	0.2738 (11)	0.267 (2)	0.2993 (11)	0.021 (6)*	
H1NB	0.2156 (14)	−0.068 (3)	0.2000 (12)	0.044 (8)*	
H2NB	0.2737 (12)	0.066 (2)	0.2531 (11)	0.024 (7)*	

### Atomic displacement parameters (Å^2^)

**Table:**

	*U*^11^	*U*^22^	*U*^33^	*U*^12^	*U*^13^	*U*^23^
S1A	0.0318 (3)	0.0154 (2)	0.0125 (2)	−0.0018 (2)	0.0023 (2)	0.00261 (19)
O1A	0.0226 (7)	0.0114 (7)	0.0162 (7)	0.0012 (6)	0.0019 (6)	−0.0002 (5)
N1A	0.0223 (9)	0.0110 (8)	0.0166 (8)	0.0014 (7)	0.0013 (7)	0.0003 (6)
N2A	0.0204 (9)	0.0111 (8)	0.0147 (8)	−0.0002 (7)	0.0040 (7)	−0.0004 (6)
C1A	0.0306 (12)	0.0164 (10)	0.0218 (11)	−0.0001 (9)	0.0074 (9)	0.0020 (8)
C2A	0.0291 (12)	0.0181 (11)	0.0412 (15)	−0.0059 (9)	0.0108 (11)	0.0011 (10)
C3A	0.0224 (12)	0.0248 (12)	0.0486 (16)	−0.0064 (10)	0.0006 (11)	−0.0074 (11)
C4A	0.0229 (12)	0.0302 (13)	0.0317 (13)	0.0015 (10)	−0.0046 (10)	−0.0034 (10)
C5A	0.0216 (11)	0.0198 (10)	0.0235 (11)	0.0003 (8)	0.0020 (9)	0.0028 (8)
C6A	0.0205 (10)	0.0166 (10)	0.0196 (10)	0.0005 (8)	0.0064 (8)	0.0002 (8)
C7A	0.0212 (10)	0.0111 (9)	0.0131 (9)	−0.0018 (8)	−0.0023 (8)	0.0000 (7)
C8A	0.0237 (10)	0.0111 (9)	0.0143 (9)	−0.0008 (8)	0.0006 (8)	0.0005 (7)
C9A	0.0231 (10)	0.0152 (9)	0.0147 (10)	−0.0017 (8)	0.0010 (8)	0.0000 (8)
C10A	0.0306 (12)	0.0176 (10)	0.0183 (10)	0.0028 (9)	0.0060 (9)	−0.0027 (8)
C11A	0.0287 (12)	0.0249 (12)	0.0286 (13)	−0.0006 (10)	0.0076 (10)	−0.0014 (10)
C12A	0.0349 (15)	0.0530 (18)	0.0464 (17)	0.0089 (13)	0.0163 (13)	−0.0037 (14)
C13A	0.0295 (14)	0.068 (2)	0.0352 (16)	0.0052 (14)	−0.0014 (12)	0.0024 (15)
S1B	0.0310 (3)	0.0189 (3)	0.0137 (2)	0.0003 (2)	−0.0030 (2)	0.00392 (19)
O1B	0.0238 (8)	0.0116 (6)	0.0149 (7)	−0.0019 (6)	−0.0016 (6)	−0.0007 (5)
N1B	0.0235 (9)	0.0116 (8)	0.0147 (8)	−0.0020 (7)	−0.0030 (7)	−0.0019 (7)
N2B	0.0240 (9)	0.0127 (8)	0.0137 (8)	−0.0005 (7)	−0.0039 (7)	0.0004 (6)
C1B	0.0217 (11)	0.0213 (11)	0.0324 (13)	0.0004 (9)	−0.0028 (10)	0.0035 (10)
C2B	0.0233 (12)	0.0331 (14)	0.0385 (14)	0.0029 (10)	0.0002 (10)	−0.0018 (11)
C3B	0.0278 (13)	0.0318 (14)	0.0496 (17)	0.0116 (11)	−0.0088 (12)	−0.0068 (12)
C4B	0.0466 (16)	0.0216 (12)	0.0532 (19)	0.0102 (12)	−0.0217 (14)	0.0029 (12)
C5B	0.0409 (14)	0.0241 (12)	0.0311 (13)	0.0023 (11)	−0.0128 (11)	0.0050 (10)
C6B	0.0235 (11)	0.0182 (10)	0.0235 (11)	0.0007 (9)	−0.0091 (9)	0.0012 (8)
C7B	0.0199 (10)	0.0136 (9)	0.0130 (9)	0.0024 (8)	0.0017 (8)	0.0006 (7)
C8B	0.0232 (10)	0.0138 (9)	0.0130 (9)	0.0005 (8)	−0.0006 (8)	0.0008 (7)
C9B	0.0219 (10)	0.0165 (9)	0.0143 (9)	0.0014 (8)	0.0017 (8)	−0.0028 (8)
C10B	0.0257 (11)	0.0221 (11)	0.0177 (10)	−0.0022 (9)	−0.0032 (9)	−0.0051 (9)
C11B	0.0246 (13)	0.0244 (14)	0.0233 (13)	−0.0004 (11)	−0.0021 (11)	−0.0031 (11)
C11C	0.0246 (13)	0.0244 (14)	0.0233 (13)	−0.0004 (11)	−0.0021 (11)	−0.0031 (11)
C12B	0.0267 (13)	0.075 (2)	0.0294 (14)	−0.0107 (14)	−0.0032 (11)	−0.0126 (14)
C13B	0.0339 (15)	0.074 (2)	0.0314 (15)	−0.0169 (15)	0.0063 (12)	−0.0169 (15)

### Geometric parameters (Å, °)

**Table:**

S1A—C8A	1.739 (2)	C1B—C2B	1.389 (3)
S1A—C6A	1.779 (2)	C1B—C6B	1.390 (3)
O1A—C7A	1.279 (2)	C1B—H1BA	0.9300
N1A—C9A	1.336 (3)	C2B—C3B	1.383 (4)
N1A—N2A	1.366 (2)	C2B—H2BA	0.9300
N1A—H1NA	0.94 (3)	C3B—C4B	1.377 (4)
N2A—C7A	1.359 (2)	C3B—H3BA	0.9300
N2A—H2NA	0.90 (2)	C4B—C5B	1.390 (4)
C1A—C2A	1.385 (3)	C4B—H4BA	0.9300
C1A—C6A	1.401 (3)	C5B—C6B	1.400 (3)
C1A—H1AA	0.9300	C5B—H5BA	0.9300
C2A—C3A	1.381 (4)	C7B—C8B	1.425 (3)
C2A—H2AA	0.9300	C8B—C9B	1.395 (3)
C3A—C4A	1.382 (3)	C9B—C10B	1.491 (3)
C3A—H3AA	0.9300	C10B—C11C	1.451 (16)
C4A—C5A	1.395 (3)	C10B—C11B	1.520 (3)
C4A—H4AA	0.9300	C10B—H10C	0.9600
C5A—C6A	1.390 (3)	C10B—H10D	0.9601
C5A—H5AA	0.9300	C10B—H10E	0.9599
C7A—C8A	1.420 (3)	C10B—H10F	0.9601
C8A—C9A	1.393 (3)	C11B—C13B	1.495 (4)
C9A—C10A	1.491 (3)	C11B—C12B	1.537 (4)
C10A—C11A	1.534 (3)	C11B—H10F	1.5675
C10A—H10A	0.9700	C11B—H11B	0.9800
C10A—H10B	0.9700	C11B—H13H	1.5112
C11A—C13A	1.520 (4)	C11C—C13B	1.452 (16)
C11A—C12A	1.524 (3)	C11C—C12B	1.680 (16)
C11A—H11A	0.9800	C11C—H10C	1.4813
C12A—H12A	0.9600	C11C—H11C	0.9800
C12A—H12B	0.9600	C11C—H13F	1.4689
C12A—H12C	0.9600	C12B—H12D	0.9600
C13A—H13A	0.9600	C12B—H12E	0.9600
C13A—H13B	0.9600	C12B—H12F	0.9600
C13A—H13C	0.9600	C12B—H12G	0.9599
S1B—C8B	1.741 (2)	C12B—H12H	0.9600
S1B—C6B	1.783 (2)	C12B—H12I	0.9601
O1B—C7B	1.284 (2)	C13B—H13D	0.9602
N1B—C9B	1.333 (3)	C13B—H13E	0.9600
N1B—N2B	1.367 (2)	C13B—H13F	0.9598
N1B—H1NB	0.95 (3)	C13B—H13G	0.9602
N2B—C7B	1.355 (3)	C13B—H13H	0.9602
N2B—H2NB	0.84 (3)	C13B—H13I	0.9598
			
C8A—S1A—C6A	104.12 (10)	C11C—C10B—H10C	72.6
C9A—N1A—N2A	109.06 (17)	C9B—C10B—H10C	108.8
C9A—N1A—H1NA	129.2 (17)	C11B—C10B—H10C	108.9
N2A—N1A—H1NA	120.6 (17)	C11C—C10B—H10D	129.5
C7A—N2A—N1A	109.86 (17)	C9B—C10B—H10D	108.7
C7A—N2A—H2NA	127.7 (15)	C11B—C10B—H10D	108.6
N1A—N2A—H2NA	121.9 (15)	H10C—C10B—H10D	107.8
C2A—C1A—C6A	119.5 (2)	C11C—C10B—H10E	107.9
C2A—C1A—H1AA	120.3	C9B—C10B—H10E	107.4
C6A—C1A—H1AA	120.3	C11B—C10B—H10E	135.9
C3A—C2A—C1A	120.8 (2)	H10D—C10B—H10E	70.2
C3A—C2A—H2AA	119.6	C11C—C10B—H10F	107.3
C1A—C2A—H2AA	119.6	C9B—C10B—H10F	107.7
C2A—C3A—C4A	119.7 (2)	C11B—C10B—H10F	74.6
C2A—C3A—H3AA	120.2	H10C—C10B—H10F	137.4
C4A—C3A—H3AA	120.2	H10E—C10B—H10F	107.1
C3A—C4A—C5A	120.6 (2)	C13B—C11B—C10B	114.1 (2)
C3A—C4A—H4AA	119.7	C13B—C11B—C12B	111.0 (2)
C5A—C4A—H4AA	119.7	C10B—C11B—C12B	107.8 (2)
C6A—C5A—C4A	119.5 (2)	C13B—C11B—H10F	146.1
C6A—C5A—H5AA	120.2	C12B—C11B—H10F	97.6
C4A—C5A—H5AA	120.2	C13B—C11B—H11B	107.9
C5A—C6A—C1A	119.9 (2)	C10B—C11B—H11B	107.9
C5A—C6A—S1A	124.09 (16)	C12B—C11B—H11B	107.9
C1A—C6A—S1A	115.95 (17)	H10F—C11B—H11B	78.8
O1A—C7A—N2A	122.10 (18)	C10B—C11B—H13H	130.6
O1A—C7A—C8A	132.11 (18)	C12B—C11B—H13H	119.7
N2A—C7A—C8A	105.74 (17)	H10F—C11B—H13H	137.3
C9A—C8A—C7A	107.20 (17)	H11B—C11B—H13H	70.8
C9A—C8A—S1A	126.62 (16)	C10B—C11C—C13B	121.3 (11)
C7A—C8A—S1A	126.10 (15)	C10B—C11C—C12B	104.0 (10)
N1A—C9A—C8A	108.13 (18)	C13B—C11C—C12B	105.6 (10)
N1A—C9A—C10A	121.20 (18)	C13B—C11C—H10C	141.3
C8A—C9A—C10A	130.58 (19)	C12B—C11C—H10C	111.3
C9A—C10A—C11A	112.67 (18)	C10B—C11C—H11C	108.4
C9A—C10A—H10A	109.1	C13B—C11C—H11C	108.4
C11A—C10A—H10A	109.1	C12B—C11C—H11C	108.4
C9A—C10A—H10B	109.1	H10C—C11C—H11C	70.6
C11A—C10A—H10B	109.1	C10B—C11C—H13F	138.3
H10A—C10A—H10B	107.8	C12B—C11C—H13F	116.2
C13A—C11A—C12A	110.4 (2)	H10C—C11C—H13F	125.4
C13A—C11A—C10A	111.8 (2)	H11C—C11C—H13F	70.1
C12A—C11A—C10A	110.1 (2)	C11B—C12B—H12D	109.7
C13A—C11A—H11A	108.1	C11C—C12B—H12D	121.6
C12A—C11A—H11A	108.1	C11B—C12B—H12E	109.5
C10A—C11A—H11A	108.1	C11C—C12B—H12E	123.9
C11A—C12A—H12A	109.5	H12D—C12B—H12E	109.5
C11A—C12A—H12B	109.5	C11B—C12B—H12F	109.3
H12A—C12A—H12B	109.5	C11C—C12B—H12F	75.2
C11A—C12A—H12C	109.5	H12D—C12B—H12F	109.5
H12A—C12A—H12C	109.5	H12E—C12B—H12F	109.5
H12B—C12A—H12C	109.5	C11B—C12B—H12G	121.1
C11A—C13A—H13A	109.5	C11C—C12B—H12G	109.7
C11A—C13A—H13B	109.5	H12D—C12B—H12G	126.7
H13A—C13A—H13B	109.5	H12F—C12B—H12G	69.3
C11A—C13A—H13C	109.5	C11B—C12B—H12H	124.3
H13A—C13A—H13C	109.5	C11C—C12B—H12H	109.2
H13B—C13A—H13C	109.5	H12E—C12B—H12H	124.4
C8B—S1B—C6B	103.90 (10)	H12F—C12B—H12H	67.2
C9B—N1B—N2B	108.82 (17)	H12G—C12B—H12H	109.5
C9B—N1B—H1NB	129.8 (18)	C11B—C12B—H12I	75.4
N2B—N1B—H1NB	120.7 (17)	C11C—C12B—H12I	109.5
C7B—N2B—N1B	110.06 (17)	H12D—C12B—H12I	67.3
C7B—N2B—H2NB	130.0 (17)	H12E—C12B—H12I	69.2
N1B—N2B—H2NB	119.0 (17)	H12F—C12B—H12I	175.2
C2B—C1B—C6B	119.9 (2)	H12G—C12B—H12I	109.5
C2B—C1B—H1BA	120.1	H12H—C12B—H12I	109.5
C6B—C1B—H1BA	120.1	C11C—C13B—H13D	117.2
C3B—C2B—C1B	120.5 (3)	C11B—C13B—H13D	109.7
C3B—C2B—H2BA	119.8	C11C—C13B—H13E	129.8
C1B—C2B—H2BA	119.8	C11B—C13B—H13E	109.8
C4B—C3B—C2B	119.8 (2)	H13D—C13B—H13E	109.5
C4B—C3B—H3BA	120.1	C11C—C13B—H13F	71.7
C2B—C3B—H3BA	120.1	C11B—C13B—H13F	108.9
C3B—C4B—C5B	120.7 (2)	H13D—C13B—H13F	109.5
C3B—C4B—H4BA	119.7	H13E—C13B—H13F	109.5
C5B—C4B—H4BA	119.7	C11C—C13B—H13G	109.3
C4B—C5B—C6B	119.5 (3)	C11B—C13B—H13G	125.2
C4B—C5B—H5BA	120.2	H13E—C13B—H13G	119.8
C6B—C5B—H5BA	120.2	H13F—C13B—H13G	76.2
C1B—C6B—C5B	119.6 (2)	C11C—C13B—H13H	110.0
C1B—C6B—S1B	123.98 (17)	C11B—C13B—H13H	72.3
C5B—C6B—S1B	116.37 (19)	H13D—C13B—H13H	76.5
O1B—C7B—N2B	121.90 (18)	H13E—C13B—H13H	63.6
O1B—C7B—C8B	132.18 (18)	H13F—C13B—H13H	172.5
N2B—C7B—C8B	105.87 (18)	H13G—C13B—H13H	109.5
C9B—C8B—C7B	106.66 (18)	C11C—C13B—H13I	109.1
C9B—C8B—S1B	126.65 (16)	C11B—C13B—H13I	121.7
C7B—C8B—S1B	126.60 (16)	H13D—C13B—H13I	127.8
N1B—C9B—C8B	108.55 (18)	H13E—C13B—H13I	46.2
N1B—C9B—C10B	120.52 (18)	H13F—C13B—H13I	63.4
C8B—C9B—C10B	130.91 (19)	H13G—C13B—H13I	109.5
C11C—C10B—C9B	119.0 (6)	H13H—C13B—H13I	109.5
C9B—C10B—C11B	113.98 (18)		
			
C9A—N1A—N2A—C7A	1.0 (2)	C4B—C5B—C6B—S1B	−177.12 (19)
C6A—C1A—C2A—C3A	1.1 (3)	C8B—S1B—C6B—C1B	−6.3 (2)
C1A—C2A—C3A—C4A	−1.5 (4)	C8B—S1B—C6B—C5B	171.51 (18)
C2A—C3A—C4A—C5A	0.8 (4)	N1B—N2B—C7B—O1B	175.70 (18)
C3A—C4A—C5A—C6A	0.3 (3)	N1B—N2B—C7B—C8B	−1.9 (2)
C4A—C5A—C6A—C1A	−0.8 (3)	O1B—C7B—C8B—C9B	−176.2 (2)
C4A—C5A—C6A—S1A	176.58 (17)	N2B—C7B—C8B—C9B	1.0 (2)
C2A—C1A—C6A—C5A	0.1 (3)	O1B—C7B—C8B—S1B	7.0 (3)
C2A—C1A—C6A—S1A	−177.48 (17)	N2B—C7B—C8B—S1B	−175.73 (16)
C8A—S1A—C6A—C5A	−3.8 (2)	C6B—S1B—C8B—C9B	108.8 (2)
C8A—S1A—C6A—C1A	173.60 (16)	C6B—S1B—C8B—C7B	−75.1 (2)
N1A—N2A—C7A—O1A	177.07 (18)	N2B—N1B—C9B—C8B	−1.4 (2)
N1A—N2A—C7A—C8A	−0.8 (2)	N2B—N1B—C9B—C10B	180.00 (18)
O1A—C7A—C8A—C9A	−177.2 (2)	C7B—C8B—C9B—N1B	0.3 (2)
N2A—C7A—C8A—C9A	0.3 (2)	S1B—C8B—C9B—N1B	176.98 (16)
O1A—C7A—C8A—S1A	5.8 (3)	C7B—C8B—C9B—C10B	178.6 (2)
N2A—C7A—C8A—S1A	−176.61 (15)	S1B—C8B—C9B—C10B	−4.7 (3)
C6A—S1A—C8A—C9A	110.8 (2)	N1B—C9B—C10B—C11C	61.5 (9)
C6A—S1A—C8A—C7A	−72.8 (2)	C8B—C9B—C10B—C11C	−116.7 (8)
N2A—N1A—C9A—C8A	−0.8 (2)	N1B—C9B—C10B—C11B	103.1 (2)
N2A—N1A—C9A—C10A	−177.72 (18)	C8B—C9B—C10B—C11B	−75.1 (3)
C7A—C8A—C9A—N1A	0.3 (2)	C11C—C10B—C11B—C13B	51.9 (10)
S1A—C8A—C9A—N1A	177.18 (16)	C9B—C10B—C11B—C13B	−55.2 (3)
C7A—C8A—C9A—C10A	176.8 (2)	C11C—C10B—C11B—C12B	−72.0 (10)
S1A—C8A—C9A—C10A	−6.3 (3)	C9B—C10B—C11B—C12B	−179.0 (2)
N1A—C9A—C10A—C11A	94.6 (2)	C9B—C10B—C11C—C13B	32.4 (16)
C8A—C9A—C10A—C11A	−81.6 (3)	C11B—C10B—C11C—C13B	−59.8 (11)
C9A—C10A—C11A—C13A	−60.0 (3)	C9B—C10B—C11C—C12B	150.8 (5)
C9A—C10A—C11A—C12A	176.9 (2)	C11B—C10B—C11C—C12B	58.6 (9)
C9B—N1B—N2B—C7B	2.1 (2)	C13B—C11B—C12B—C11C	−63.0 (10)
C6B—C1B—C2B—C3B	0.2 (4)	C10B—C11B—C12B—C11C	62.7 (10)
C1B—C2B—C3B—C4B	1.0 (4)	C10B—C11C—C12B—C11B	−65.9 (10)
C2B—C3B—C4B—C5B	−1.4 (4)	C13B—C11C—C12B—C11B	62.8 (9)
C3B—C4B—C5B—C6B	0.5 (4)	C10B—C11C—C13B—C11B	60.7 (12)
C2B—C1B—C6B—C5B	−1.1 (3)	C12B—C11C—C13B—C11B	−56.9 (8)
C2B—C1B—C6B—S1B	176.58 (18)	C10B—C11B—C13B—C11C	−51.2 (10)
C4B—C5B—C6B—C1B	0.8 (3)	C12B—C11B—C13B—C11C	70.9 (10)

### Hydrogen-bond geometry (Å, °)

**Table:**

*D*—H···*A*	*D*—H	H···*A*	*D*···*A*	*D*—H···*A*
N1A—H1NA···O1A^i^	0.94 (3)	1.71 (3)	2.639 (2)	171 (3)
N2A—H2NA···O1B	0.90 (2)	1.88 (3)	2.752 (2)	161 (2)
N1B—H1NB···O1B^ii^	0.95 (3)	1.67 (3)	2.619 (2)	176 (3)
N2B—H2NB···O1A	0.85 (3)	1.91 (3)	2.731 (2)	162 (2)
C10B—H10D···S1A^iii^	0.96	2.84	3.721 (2)	153

Symmetry codes: (i) −*x*+1/2, *y*+1/2, *z*; (ii) −*x*+1/2, *y*−1/2, *z*; (iii) *x*, −*y*, *z*−1/2.
